# More about Quacks

**Published:** 1906-01

**Authors:** 


### EDITORIAL NOTES AND COMMENTS.

The Business office of THE JOURNAL-RECORD is No. 1007-1008 Century Building.

The Editorial office is 318-319-320 The Prudential.

Address all Business communications to Dr. M. B. Hutchins, Mgr.

Make remittances payable to THE ATLANTA JOURNAL-RECORD OF MEDICINE.

On matters pertaining to the Editorial and Original Communications address Dr. Bernard Wolff, Atlanta.

Reprints of original articles will be furnished at cost price. Requests for the same should always be made *on the manuscript*.

We will present, post-paid, on request, to each contributor of an original article, twenty (20) marked copies of THE JOURNAL-RECORD containing such article.

### MORE ABOUT QUACKS.

THE New York Medical Society has adopted the following resolutions directed against quacks and quackery:

"WHEREAS, The Medical Society for over one hundred years, with the sanction of the State of New York, has been engaged in an effort to "regulate the practice of physic and surgery," and

WHEREAS, The Legislature of said State has expressly authorized the enforcement by this and other county medical societies in the State of the public health law regulating said practice, and

WHEREAS, Hundreds of prosecutions and convictions have resulted as a direct outcome of the work of this Society in enforcing the law, and

WHEREAS, A large percentage of the quacks and charlatans convicted as a result of the work of this Society have been freely admitted to the advertising columns of various papers in the city and county of New York, and the protection of the public health been made much difficult as a result of said advertisement; now, therefore, be it

*Resolved*, That the Medical Society of the County of New York, at its hundredth annual meeting, emphatically protest against this criminal alliance between quacks and certain newspapers in the city of New York and elsewhere; that in the courts of honor and conscience and morals, if not n the courts of law, the newspaper that profits by the publication of the alluringly false advertisements of such notorious quacks and charlatans as William H. Hale, Henry H. Kane, L. R. Williams and many others of similar ilk, whose filthy advertisements can be found in prominent places in the advertising columns of certain New York papers, are in no wise less guilty than these charlatans themselves. Be it further

*Resolved*, That the thanks of this Society is due to the *Ladies' Home Journal* and *Collier's Weekly* for their telling exposures of the criminal alliance existing not only between quacks and some newspapers, but between powerful companies controlling well-known patent medicines long since proven to be dangerous to the health and comfort of the people.

It is a fine commentary that two lay papers can accomplish more against the evil of quackery than the combined medical press, a commentary on the real impotence of the medical profession to cope with the evil or to mitigate it by all of its fervid denunciations, anathemas and broadsides. The deduction from this all too evident fact is that the public has no confidence in the doctor when he attacks quackery, but listens indulgently with the mental comment that perhaps the doctor is a bit of a quack himself. Even if the lay papers take up the matter and fight the infliction what will be the result? There will be a suppression here and a compensatory dilatation there. As well try to drown a puppy by holding him under with a straw. The real fault is with the people. They are resolute in one thing, however vacillating in others, and that is the exercise of their ancient privilege to diagnose their own ills and physic themselves. One encounters very few people indeed who have not at one time used Coke's Dandruff Cure, the Pyramid Pile Cure or a one-night corn cure, to select a few favored localities of the body very adaptable to easy diagnosis. There are more boys than one may conjecture who have hoarded their pennies to purchase a bottle of manhood restorer to offset the effects of a certain substitutive act in the sphere of pubescent libidiny. Quack medicines like other commodities are regulated by supply and demand. So long as there is a demand for them they will flourish, law or no law. If the demand cease, they will go out of business. So long as the reverend brother who draws tears at the graphic depiction of the drunkard's home or the death of the rum maniac desires his "grain'' in the form of Peruna, he is very apt to get it, especially as his church paper so cordially recommends it. The poor devil with disseminated cancer will purchase Dr. Bye's soothing oils with the last dollar of mortgage because the "doctor" says it will cure him and other doctors say they can not. That eminent scientist surrounded as with the halo of a saint by the incense from his retorts and percolators can surely touch that weak spot in Henderson's lungs and rid him by a simple and natural process of the handful of tubercle bacilli which have got into his "system." All of these things are so evident that it is almost needless to speak of them. But they are in the way of the disappearance of the quack and his nostrums, and the Medical Society of New York will doubtless have another chance to adopt similar resolutions on its second centenary. The fight begins wrong and there is not much chance of it ending right. Is it proper to attack the product of the quack and let him escape? Is it worth while to expose the nostrums advertised in the newspapers and allow the organized bands of irregulars and dangerous sectarians to go about their business and ply their menacing avocation without molestation? Abortionists have become a public convenience as essential as blind tigers and bawdy houses, and every one knows how necessary an adjunct these are to civilization. There are probably very few thriving towns which do not support one of these love's labor lost institutions. The slaughter of the innocents goes merrily on while Lydia Pinkham gets some hot shot from *Collier's* and the *Homely Ladies' Journal* and the statement of actual percentage of alcohol in Peruna gives spasms of indignation to a deceived and outraged public. And the shambling throng of healers of various cults trail not very far behind the philanthropic census discouragers. The public is being aroused from a fatuous indolence in the crusade against tuberculosis, but syphilis is still an unmentionable quantity in the matter of reform, and the little giant, gonoccoccus, is busily at work over his cheerful task of wrecking health and happiness. There is much that is commendable in the valiant fight a few of the lay publications are waging against the shams and frauds of charlatanism and we doff our hat to them with respect and admiration. But we call attention, as matters urgently requiring moral sanitation, to the middens and cesspits, whose mephitic vapors alone they are attempting to neutralize and disinfect while the bubbling mass of gray filth underneath is left to seethe and "work" undisturbed. If the papers would begin at this end of the problem and work forward the dawn of hope would soon appear in the purple East.

